# Spanish Version of the Scale “Eventos Adversos Associados às Práticas de Enfermagem” (EAAPE): Validation in Nursing Students

**DOI:** 10.3390/nursrep12010012

**Published:** 2022-02-14

**Authors:** Antonio Martínez-Sabater, Carlos Saus-Ortega, Mónica Masiá-Navalon, Elena Chover-Sierra, María Luisa Ballestar-Tarín

**Affiliations:** 1 Nursing Department, Facultat d’Infermeria i Podologia, Universitat de València, 46010 València, Spain; antonio.martinez-sabater@uv.es (A.M.-S.); m.luisa.ballestar@uv.es (M.L.B.-T.); 2 Nursing Care and Education Research Group (GRIECE), GIUV2019-456, Nursing Department, Universitat de Valencia, 46010 València, Spain; sausor@uv.es; 3 Grupo Investigación en Cuidados (INCLIVA), Hospital Clínico Universitario de Valencia, 46010 València, Spain; 4 Nursing School “La Fe”, Generalitat Valenciana, 46026 València, Spain; 5 Hospital de la Ribera, 46600 València, Spain; monicamasia@hotmail.es; 6 Internal Medicine, Consorcio Hospital General Universitario de Valencia, 46014 València, Spain

**Keywords:** patient safety, nursing, students, quality of healthcare, safety management

## Abstract

Healthcare carried out by different health professionals, including nurses, implies the possible appearance of adverse events that affect the safety of the patient and may cause damage to the patient. In clinical practice, it is necessary to have measurement instruments that allow for the evaluation of the presence of these types of events in order to prevent them. This study aims to validate the “Eventos adversos associados às práticas de enfermagem” (EAAPE) scale in Spanish and evaluate its reliability. The validation was carried out through a cross-sectional study with a sample of 337 nursing students from the University of Valencia recruited during the 2018–19 academic year. An exploratory factor analysis was carried out using principal components and varimax rotation. The factor analysis extracted two factors that explained 32.10% of the total variance. Factor 1 explains 22.19% and refers to the “adverse results” of clinical practice (29 items), and factor 2 explains 9.62% and refers to “preventive practices” (24 items). Both factors presented high reliability (Cronbach’s alpha 0.902 and 0.905, respectively). The Spanish version of the EAAPE is valid and reliable for measuring the perception of adverse events associated with nursing practice and the presence of prevention measures.

## 1. Introduction

Due to the characteristics of healthcare, patient safety is considered a fundamental element that must be an essential objective of healthcare systems and must also be considered a transversal dimension [1,2]. The presence of adverse events (AE) associated with healthcare (as with most human actions) is proven. However, it was not until the end of the 20th century that research studies showed the high prevalence of safety problems that make clear the need to promote policies, programs, and strategies to improve patient safety [1,3]. In Spain, the strategy for the Quality Plan of the National Health System aims to “Improve the safety of patients treated in the health centers of the National Health System,” trying to promote research on patient safety [2,4,5,6]. 

Patient safety culture is defined as a pattern of behavior between a human being and a unified organization, based on beliefs and values, striving for the continuous reduction of AE in the care of patients. It should become a commitment for professionals and a priority in health management. The World Health Organization (WHO) considers the “culture of safety” as one of the relevant human factors for patient safety. This culture of safety can be improved with effective teamwork and organizational learning, affecting aspects such as quality of care, the implementation of safe practices, and the reduction of hospital stays, among others [7,8,9].

In the context of health organizations, adverse events are defined as unintended injuries or complications that derive from healthcare rather than from the patient’s clinical situation and that can cause death, disability at the time of discharge, or a prolonged hospital stay [10]. It should be taken into account that the occurrence of AE is considered a public health problem that reflects an impairment in the quality of healthcare with repercussions at different levels and that can be quantitatively detected [11]. This situation has raised growing concern about the need to research and sensitize professionals towards this issue. Different studies have quantified their incidence as a consequence of healthcare as reaching up to 6.8%. This incidence is higher in older people and supposes more extended hospital stays and higher morbidity, having important repercussions on the average cost of care and the increase in hospital admissions [12,13]. The AE associated with healthcare practice identified in related studies in different healthcare settings are diverse: AE related to care (32.4%), procedures (32.4%), medication (13.5%), nosocomial infections (10.8%), and diagnostic errors (8.1%). In the different studies, it is established that between 50 and 63.2% of events are considered avoidable [10,14,15]. It is also noteworthy that these AE affect patients and can also have a tremendous economic impact on the health system [16] as well as cause damage to professionals, known as second victim syndrome [17,18].

In Spain, different studies have been published to quantify AE in different settings such as hospitals (ENEAS) [19], primary care (APEAS) [20,21], social health (EARCAS) [22], intensive medicine units (SYREC) [23], and emergency services (EVADUR) [24]. All of these studies have established high numbers of AE. The notification of AE is an essential aspect of improving the safety and quality of services and allowing people and institutions to learn, prioritizing strategies. For this, the development or validation of instruments that allow for accurate monitoring of AE at all levels of care is recommended as a priority line of research [25,26]. However, one of the problems identified in the literature regarding patient safety is under-reporting and the lack of safety culture on the part of professionals [17]. For this reason, it is necessary to promote the use of different notification systems that signify the beginning of the analysis process for the subsequent implementation of improvement strategies [27].

Due to the characteristics of their work, nurses acquire a fundamental role in the maintenance and promotion of patient safety. The objective of nursing care is to ensure the best possible results in the clinical condition of patients and the lowest rates of complications when performing procedures. Nurses face safety problems inherent to their discipline, leading to AE associated with their practice [7,28]. Various investigations [10,11,25] relate these AE not only to organizational factors or to characteristics of the patient himself but also to aspects of nursing work environments, such as leadership, the organizational structure of work, academic level, the presence of burnout, and the burden of teamwork in nursing. AE related to nursing practice include drug preparation or administration errors, pressure ulcers, and injuries related to accidental falls [29,30,31].

The safety culture that must permeate all care activity implies that nursing students must also receive training in this line to incorporate skills, knowledge, and attitudes that will allow them to improve their future practice, improving quality and patient safety in the clinical setting [32].

This work was proposed due to the need identified in previous studies to have valid and reliable instruments that allow for the analysis of AE associated with healthcare [33]. This analysis focused on the evaluation through retrospective designs of AE that have already occurred, which is the type that appears most frequently in studies that analyze events related to healthcare [34].

Among the validated instruments for analyzing AE with nursing activity, the “Eventos adversos associados às práticas de enfermagem” (EAAPE; Adverse Events Associated with Nursing Practice) scale stands out [35,36]. The advantage of this instrument over others is that it allows for the evaluation of preventive measures to avoid possible AE, and it does not focus exclusively on the analysis of these events after their appearance.

The construction of this scale was carried out following the quality model proposed by Donabedian for the structure, processes, and results and is based on Reason’s explanatory model of causality of AE. The original version is an instrument for the diagnosis of AE that, in turn, according to its authors, “serves to sensitize nursing professionals of the role they play in improving patient safety.” The scale has a total of 55 items divided into two subscales. Subscale 1 is called “nursing practices”, and it is made up of 42 items that detail aspects of the nursing care process. Subscale 2, called “risk and occurrence of adverse events,” has 13 items that refer to the possible adverse results of nursing intervention. A five-point Likert-type scale is used, where the level of agreement or disagreement with each item is specified. The reliability of the nursing practices subscale, Cronbach’s alpha, was 0.90. The internal consistency of subscale 2, risk and occurrence of adverse events, obtained a Cronbach’s alpha of 0.85 [35].

The main objective of this work is the validation into Spanish of the EAAPE and the evaluation of its psychometric properties.

## 2. Materials and Methods

### 2.1. Design

A descriptive, cross-sectional, multicenter study was developed to evaluate the reliability, factorial structure, and internal consistency of the Spanish version of the EAAPE questionnaire.

### 2.2. Adaptation and Translation into the Spanish Language

The EAAPE, as already mentioned, is a diagnostic instrument that assesses the frequency of nursing processes and practices associated with patient safety. The original scale was made by Castilho [35]. To carry out this study, the transcultural translation and adaptation process was carried out according to the multiphase interactive model (based on the traditional translation–back-translation method). The original language of this scale is Portuguese, so it was necessary before this pilot study among nursing students to carry out a linguistic validation that included the processes of direct translation, back-translation, and revision by a multidisciplinary committee of both versions (translated and back-translated) to guarantee the maximum intercultural equivalence.

### 2.3. Sample

The sample participating in the piloting of the Spanish version of the EAAPE consisted of 337 students from the Faculty of Nursing and Podiatry of the University of Valencia. This represents a very high percentage (96.28%) of the 350 students enrolled in the fourth-year internship subjects. All of them were in their last year of their nursing degree; therefore, they were finishing their training period and about to start their professional career. They were doing internships in different public hospitals of the Valencian Community in different services: medical, surgical, or special.

Non-probabilistic convenience sampling was used, although sufficient sample size was achieved to carry out the piloting of the instrument. Following the criteria of authors such as Martinez-Arias [37,38], it was considered that there should be between 5 and 10 participants per item on the scale, which in our case meant a sample of at least 275 participants.

### 2.4. Data Collection

Students were invited to participate in the study, taking advantage of their attendance at the scheduled lessons. The data collection instrument was designed with the help of Google Forms. It consisted of two parts, the first that collected data to characterize the participants and the second part with the Spanish version of the EAAPE. A full explanation of the study was provided through an information sheet at the beginning of the form. Those students who agreed to participate signed the informed consent before accessing the questionnaire. The completed questionnaires were identified with an alphanumeric code to guarantee the confidentiality of all the data collected.

### 2.5. Data Analysis

For the descriptive analysis of the study data, means and standard deviation were used for quantitative variables and frequencies and percentages for qualitative variables. For the psychometric validation, the study of the structure was carried out with exploratory factor analysis (EFA) of principal components, with varimax rotation for each subscale as in the original scale, determining the Eigen load value for each item, the percentage of variance explained, and relevant validity. To check the adequacy of the analysis, the correlation matrix, Bartlett’s sphericity test, and the Kaiser–Meyer–Olkin (KMO) statistic were used.

Next, the reliability of the questionnaire was evaluated through the analysis of its internal consistency by calculating the Cronbach’s alpha coefficient of both the global scale and the two factors or subscales extracted, considering as a good a priori criterion an alpha equal to or greater than 0.70.

The data were analyzed using IBM SPSS Statistics for Windows, Version 26.0. (Armonk, NY, USA: IBM Corp), and a level of statistical significance of 0.05 was considered for all the analysis.

### 2.6. Ethical Considerations

As the study was carried out in the environment of the University of Valencia and health centers associated with it through a clinical practice agreement, the indications of the University of Valencia on the non-need for approval by an ethics committee were followed because it is an opinion survey about a topic or issue, professional status, or satisfaction with certain matters.

However, a preamble was included in the survey stating that it belonged to a research project, including its purpose and the benefits this information may provide, the willingness of the participation, the anonymous treatment of data, and a reference to the processing of information according to the applicable Data Protection Law. This preamble included a paragraph in which the survey respondent voluntarily accepts participation in the research and gives consent tacitly when responding to the survey.

## 3. Results

### 3.1. Participants Features

The mean age of the 337 participants was 22.22 years (SD 4.00). Table 1 shows the rest of the characteristics of the participants. These students take the Practicum III and Practicum IV subjects during the first and second semesters of the academic year in different services of the public hospitals that have an agreement with the Faculty of Nursing of the University of Valencia.

### 3.2. Results Obtained in the EAAPE 

Table 2 shows the results obtained by the participants in the EAAPE questionnaire, as percentages achieved in each category of responses. 

### 3.3. Differences in the Responses to the Questionnaire According to Internship Centers and Hospitalization Units

Table 3 shows the differences identified in the students’ responses to the questionnaire depending on the unit and hospital in which they developed their activity. These differences are only in some cases statistically significant.

### 3.4. Factor Structure of the Spanish Version of the EAAPE

Both the results of the Kaiser–Meyer–Olkin (KMO) test (with a value of 0.819) and Bartlett’s sphericity test (with a value of *p* < 0.05) show the relevance of performing factor analysis.

The EFA shows us a two-factor structure, which explains 32.1% of the total variance. The first factor explains 22.186% of the total variance, and the second factor explains 9.925%.

Taking as a criterion to assign an item to the factor in which it had a factor load or saturation greater than 0.30, factor I grouped 29 items related to the appearance of AE that affect patient safety. This factor was called “Adverse outcomes” because it showed the consequences of inadequate practice on the part of nurses.

In factor II, 24 items related to aspects that favor the prevention of AE that may affect patient safety were grouped. For this reason, this factor has been called “Preventive Nursing Practices.”

Table 4 shows the factor load matrix of each item for the two-factor structure of each item with both factors.

### 3.5. Reliability

The reliability analysis shows adequate values of the Cronbach index, both for the global instrument (Cronbach’s alpha = 0.801) and for each of the two subscales that compose it: adverse outcomes (Cronbach’s alpha = 0.902) and preventive nursing practices (Cronbach’s alpha = 0.905).

## 4. Discussion

In this study, the psychometric properties of the Spanish-language version of an instrument for evaluating AE associated with nursing practices called EAAPE, initially developed in Portuguese, have been validated and analyzed.

This work was proposed with a sample of 337 students from the Faculty of Nursing and Podiatry of the University of Valencia while doing their internships in different hospitals and healthcare units. Different studies on the notification of AE by students during their training show this ability to identify and report AE related to nursing practice [39,40]. In addition, identifying these AE (or their possible occurrence, and therefore, seeking strategies to prevent them) can involve a process of reflection on their practice and generate improvements in academic results [39,41,42]. Likewise, these students had carried out practices in simulated environments before their contact with healthcare centers. This training in simulated environments allows for the acquisition of competencies on patient safety in a very similar way to how they are acquired in actual clinical practice scenarios [43,44,45].

Reflection on the practical activity itself allows real errors and/or possible risk situations to be identified. The students of our university have been trained for this during their degree, even through active methodologies such as clinical simulation, so that they can develop this capacity for critical reflection.

The Spanish version of the EAAPE validated in this work consists of two subscales, much like the original instrument [35]. The subscale called “adverse outcomes” is made up of 29 items, and the subscale “Preventive nursing practices” is made up of 24 items. The reliability analysis of the instrument yielded high values both for the overall questionnaire and for each of the two subscales (similar to those obtained by Castilho).

The subscale “adverse outcomes” allows us to show the perceptions of students about the frequency of the occurrence of AE associated with nursing practice (falls, medication errors, ulcers, and infections). Different studies have assessed the occurrence of AE associated with nursing practices in different settings [46,47,48,49].

The students’ perception of the actual appearance of AE in the places where they carried out their practices may reflect the low incidence of these phenomena. The students participating in this study reported that there were “never or rarely” falls in 81% (item Ca 5), pressure ulcers in 41% (item Ulc 9), medication errors in 83% (item Med 2), and infections in 57% (item Inf 2).

However, regarding the risk perceived by the students about the appearance of these phenomena, they perceived the risk of falls 36.7% “sometimes,” 18.5 “frequently,” and 4.2% “always.” Regarding the risk of pressure ulcers’ appearance, 29.3% of the students perceived it “sometimes,” 28.4% “frequently,” and 14.3% “always.” Regarding the perceived risk of medication errors, 32.6% perceived it “sometimes,” 8.6% “frequently,” and 4% “always.” Likewise, regarding the risk of infections, 43.1% of the students perceived it” sometimes,” 18% “frequently,” and 3.1 “always.”

This perception of the presence of risk situations for patients associated with nursing practice reflects how training in safety culture can be an instrument that helps identify the presence of avoidable situations along with the subsequent implementation of preventive measures to avoid or reduce the appearance of AE [15,30,39,50].

The perception of the incidence of hospital accidents and the secondary effects of patients has already been used in different studies. Thus, the one International Hospital Outcome Study in 711 hospitals in five different countries found that medication errors during the previous year were perceived by 15.7%, 19.3%, and 5.1% of nurses, and nosocomial infections by 34.7%, 33.0%, and 27.9% in the US, Canada, and Germany, respectively. In addition, patient falls were perceived by 20.4% in the US, 27.9% in Canada, and 15.0% in Germany [51].

The “preventive practices” subscale assesses students’ perception of the existence of measures in practice centers that can avoid or minimize the negative effect of nursing interventions on patients. The participants in the study identify the performance of this type of measure in the different healthcare units, which makes them, as we have mentioned, suitable external evaluators.

Regarding the answers given to the items of the “preventive practices” subscale, it can be observed that 36.3% say that risk assessment scales of developing pressure ulcers are used “frequently” or “always.” In addition, 54.4% say that measures are implemented to avoid the appearance of pressure ulcers depending on certain risk factors. Other aspects of these preventive practices are reducing the risk of nosocomial infection. Thus, we see that most of the students state that hand washing is carried out properly before and after each contact with the patient (45%), after contact with body fluids (79.9%), and before performing aseptic techniques (73.6%). Additionally, more than half of students opine that handling sharps material is adequately done “frequently” (33.6%) or “always” (38.7%).

Some studies describe differences in the prevalence of AE according to specific characteristics of health centers, which include aspects such as the nurse/patient ratio, working conditions (higher ratio of nurse-to-patient and poor work environments represent an increase in the incidence of AE) [13,30,52,53], or size of the center (which may imply differences in the technification of healthcare processes and higher probability of AE) [10,54]. In addition, it has also been identified that the highest rates of AE occur in special or technical units such as ICUs, and emergency and surgical services [49,55,56,57]. These differences in the perception of the appearance of AE have also been found in this study by identifying differences in the perception of the appearance of AE and in the identification of measures to reduce the risk of appearance of AE depending on the center or the service in which they carried out their practices. Nursing students are in contact with AE related to these practices in their care practices [28,42]. That’s the reason why patient safety should be promoted in students in multiple ways, especially simulation-based training, to strengthen safe nursing behavior and reduce the incidence of AE [43,44,45].

Few studies evaluate the presence of preventive activities/knowledge of preventive activities to avoid the occurrence of AE, such as those that allow us to evaluate EAAPE. Future efforts in patient safety should be directed at this prevention line and not at identifying problems and the subsequent retrospective analysis. Together with the improvement and implementation of transformational leadership measures, this aspect may also allow for greater satisfaction of nursing staff with their care practice since it will allow them to reflect on the secondary effects of their interventions and how to avoid them [11,25,26,58].

This reflection on practice is associated with a higher level of training. Along these lines, different studies suggest that higher education in nursing is associated with lower mortality risks and a lower frequency of AE, making it necessary to investigate the relationships between nurse training and benefits [39]. Thus, higher levels of nursing staff, higher education in registered nursing, and more supportive work environments, while reducing burnout among nurses, are associated with lower rates of AE [13,29,46,52,56,59]. Training in safety culture through continuous training courses or even master’s level training will train them for such critical reflection, will improve the identification of AE, and will reduce their incidence.

As a limitation of the study, it should be noted that in this work, only the analysis carried out in the hospital setting was taken into account, not having considered areas of action such as primary care or social health.

Only students have been considered at the given time, and it is impossible to assess whether there is a better perception or implementation of the safety culture based on the development of the practice.

Finally, regarding the size and characteristics of the sample, the study has focused on students from a university center, so this perception should be assessed in different study plans.

At the same time, as an option for the future, it is proposed to compare the students’ perception with the actual data on the incidence of the appearance of AE to assess whether the students’ perception is in line with the reality of the environment.

## 5. Conclusions

The Spanish version of the EAAPE validated with this work is an instrument with adequate reliability and validity indices which can be used in our country to evaluate safe practices in the daily activities of nurses. It can be used as a starting point for strategies to improve patient safety and the quality of nursing care.

## Figures and Tables

**Table 1:** Characteristics of the participants (*n* = 337).

	*n*	%
Gender		
Male	67	19.9
Female	263	78.0
Hospital		
Hosp. Clínico	103	30.6
Hosp. General	113	33.5
Hosp. Dr. Peset	69	20.5
Hosp. La Fe	27	8.0
Other	12	3.6
Internship area		
Medical units	90	26.7
Surgical units	88	26.1
Special units	125	37.1

**Table 2:** Distribution of responses (percentages) in each category of EAAPE.

Category	Item	Never	Hardly Ever	Sometimes	Frequently	Always
Surveillance/Clinical monitoring and tracking	Vig 1	0.3	1.5	20.1	52.1	26.0
	Vig 2	0	0	14.4	61.7	24.0
	Vig 3	9.8	52.7	26.5	8.6	2.4
	Vig 4	14.4	55.4	19.5	8.1	2.7
Defense of patient rights	Def 1	0.9	9.6	37.8	39.6	12.0
	Def 2	3.0	17.2	41.3	31.9	6.6
	Def 3	0.3	7.5	22.8	40.5	28.8
	Def 4	0	4.5	13.2	44.6	37.7
	Def 5	18.4	31.1	28.7	18.7	3.0
	Def 6	21.3	54.6	19.5	4.0	0.6
	Def 7	27.9	48.2	19.7	3.6	0.6
Falls	Ca 1	21.2	27.9	16.4	19.4	15.2
	Ca 2	14.4	19.9	22.1	28.5	15
	Ca 3	7.6	16.6	23.9	33.9	17.7
	Ca 4	6.1	34.5	36.7	18.5	4.2
	Ca 5	38.4	42.6	14.1	4.8	0
Pressure ulcers	Ulc 1	7.2	10.9	16.8	26.5	38.6
	Ulc 2	9.0	19.4	21.6	26.5	23.5
	Ulc 3	22.0	18.9	22.7	15.5	20.8
	Ulc 4	7.0	12.2	26.3	38.8	15.6
	Ulc 5	5.0	13.6	22.3	40.2	18.9
	Ulc 6	2.5	6.5	13.0	45.7	32.3
	Ulc 7	2.8	14.7	22.5	37.2	22.8
	Ulc 8	5.5	22.0	29.3	28.4	14.3
	Ulc 9	12.6	28.5	33.4	23.3	2.1
Medication	Med 1	6.8	48.6	32.6	8.9	4
	Med 2	23.4	59.9	13.2	3.3	0.3
	Med 3.1	18.8	35.8	29.3	12.8	3.3
	Med 3.2	10.9	17.6	24.8	37.3	9.4
	Med 3.3	24.5	42.4	20.6	11.2	1.2
	Med 3.4	25.7	47.7	19.3	6.7	0.6
	Med 3.5	6.3	29.2	37.3	23.8	3.3
	Med 3.6	10.8	51.4	27.6	8.7	1.5
	Med 4.1	25.7	47.7	19.3	6.7	0.6
	Med 4.2	11.4	40.8	31.2	15	1.5
	Med 4.3	19.9	43.1	27.7	8.7	0.6
	Med 4.4	18.7	47.1	26.6	7	0.6
	Med 4.5	30.4	53.3	10.8	4.8	0.6
	Med 4.6	36.9	45.4	14.6	2.4	0.6
	Med 4.7	36.6	50.8	10.3	2.3	0
	Med 5.1	17.4	57.3	18.9	5.2	1.2
	Med 5.2	19.2	55.3	19.5	5.7	0.3
Healthcare-associated infection (HAIs)	Inf 1	4	31.8	43.1	18	3.1
	Inf 2	12.6	44.3	31.1	10.8	1.2
	Inf 3.1	9.6	20.4	25.1	20.7	24.3
	Inf 3.2	1.8	8.4	16.2	29.9	43.7
	Inf 3.3	1.8	7.3	10.9	24.3	55.6
	Inf 4	1.9	8.4	25.4	41.5	22.9
	Inf 5	1.8	9.3	16.5	33.6	38.7
	Inf 6	0.6	4.2	5.4	18.3	71.5
	Inf 7	1.8	6.4	14.6	29.8	47.4
	Inf 8	1.5	6.3	13.9	34.7	43.5
	Inf 9	1.2	6.4	11	29.1	52.1
General perception	P.G 1	11.3	45.1	25.4	11.3	6.9
	P.G 2	0.6	6.9	18	37.2	37.2

Note: The coding of each of the items that make up the questionnaire (and thus its full text) is presented in Supplementary Material Table S1 together with the Portuguese and Spanish versions of the instrument and an English translation (there is no validated English version of the EAAPE).

**Table 3:** Differences in the responses to the questionnaire according to internship hospital or unit.

Category	Item	Hospital		Unit	
		Chi-Sq	*p*-Value	Chi-Square	*p*-Value
Surveillance/Clinical monitoring and tracking	Vig.1	10.516	0.838	27.425	0.001 **
	Vig 2	5.296	0.725	9.695	0.046 *
	Vig 3	23.428	0.103	6.796	0.559
	Vig 4	13.865	0.609	7.352	0.499
Defense of patient rights	Def 1	10.012	0.866	4.499	0.810
	Def 2	21.546	0.158	8.586	0.378
	Def 3	13.915	0.605	6.512	0.590
	Def 4	14.681	0.259	3.726	0.714
	Def 5	21.052	0.177	7.527	0.481
	Def 6	11.069	0.865	8.736	0.365
	Def 7	19.656	0.236	10.929	0.206
Falls	Ca 1	24.595	0.077	11.803	0.160
	Ca 2	32.874	0.008 **	8.905	0.350
	Ca 3	23.291	0.106	3.353	0.910
	Ca 4	15.511	0.488	16.745	0.033 *
	Ca 5	15.671	0.207	4.438	0.618
Pressure ulcers	Ulc 1	23.804	0.094	16.347	0.038 *
	Ulc 2	15.878	0.462	18.929	0.015 *
	Ulc 3	31.440	0.012 *	16.562	0.035 *
	Ulc 4	24.894	0.072	12.219	0.142
	Ulc 5	13.039	0.670	15.896	0.044 *
	Ulc 6	11.156	0.800	18.622	0.017 *
	Ulc 7	21.903	0.146	9.769	0.282
	Ulc 8	28.235	0.030 *	25.057	0.002 **
	Ulc 9	17.695	0.342	20.108	0.010 *
Medication	Med 1	15.659	0.477	3.881	0.868
	Med 2	27.129	0.040*	1.238	0.975
	Med 3.1	12.271	0.725	4.874	0.771
	Med 3.2	16.042	0.450	5.419	0.712
	Med 3.3	56.542	0.001 **	29.810	0.001 **
	Med 3.4	44.813	0.001 **	9.099	0.334
	Med 3.5	18.003	0.324	14.292	0.074
	Med 3.6	24.092	0.088	12.299	0.138
	Med 4.1	31.499	0.012 *	3.773	0.87
	Med 4.2	25.021	0.069	7.299	0.505
	Med 4.3	19.347	0.251	17.249	0.028 *
	Med 4.4	21.378	0.164	6.444	0.598
	Med 4.5	8.832	0.920	4.415	0.818
	Med 4.6	12.052	0.740	5.674	0.684
	Med 4.7	15.965	0.193	2.758	0.839
	Med 5.1	17.207	0.372	6.499	0.592
	Med 5.2	12.702	0.694	9.983	0.125
Healthcare-associated infection (HAIs)	Inf 1	16.678	0.407	17.546	0.025 *
	Inf 2	16.694	0.406	20.279	0.009 **
	Inf 3.1	17.448	0.357	33.153	0.001 **
	Inf 3.2	35.952	0.003 **	14.297	0.074
	Inf 3.3	26.820	0.044 *	15.890	0.044 *
	Inf 4	21.965	0.144	9.489	0.303
	Inf 5	16.829	0.397	7.420	0.492
	Inf 6	21.044	0.177	9.889	0.273
	Inf 7	13.711	0.620	5.697	0.681
	Inf 8	19.754	0.232	3.757	0.878
	Inf 9	18.303	0.306	14.208	0.077
General perception	P.G 1	11.888	0.752	8.705	0.368
	P.G 2	29.791	0.019 *	9.882	0.237

Note: The coding of each of the items that make up the questionnaire (and thus its full text) is presented in Supplementary Materials 1 together with the Portuguese and Spanish versions of the instrument and an English translation (there is no validated English version of the EAAPE). * significant at level 0.05, ** significant at level 0.001

**Table 4:** Factor load matrix.

Item	Factors	
	I (Adverse Outcomes)	II (Preventive Nursing Practices)
Med 4.7	0.667	−0.114
Med 3.6	0.608	−0.194
Med 4.6	0.604	−0.239
Def 7	0.602	−0.210
Med 4.1	0.592	−0.150
Med 4.3	0.584	−0.142
Med 4.4	0.576	−0.109
Med 3.5	0.569	−0.078
Med 3.2	0.566	0.092
Med 2	0.562	−0.175
Med 4.2	0.551	−0.182
Def 6	0.538	−0.322
Ca 5	0.537	0.022
Vig 4	0.537	−0.281
Ulc 9	0.526	0.057
Med 5.2	0.526	−0.310
Med 5.1	0.517	−0.233
Med 3.1	0.514	0.144
Inf 2	0.514	−0.150
Med 4.5	0.491	−0.166
Med 3.4	0.480	−0.007
Vig 3	0.449	−0.245
Ulc 8	0.448	−0.026
Med 1	0.417	−0.178
Ca 4	0.396	−0.174
P.G.1	0.390	0.000
Def 5	0.372	−0.039
Med 3.3	0.37	−0.06
Inf 1	0.351	−0.115
Ulc 3	0.196	0.65
Ca 1	0.143	0.637
Ulc 2	0.112	0.727
Ulc 1	0.101	0.673
Ulc 4	0.038	0.754
Ca 2	−0.008	0.716
Ca 3	−0.069	0.684
Ulc 5	−0.072	0.783
Inf 5	−0.097	0.401
Ulc 6	−0.152	0.547
Def 1	−0.169	0.482
Def 3	−0.170	0.482
Inf 9	−0.196	0.375
Ulc 7	−0.199	0.540
Inf 3.1	−0.200	0.516
Inf 8	−0.230	0.487
Inf 3.2	−0.236	0.489
Inf 6	−0.238	0.404
Def 4	−0.246	0.496
Vig 1	−0.248	0.504
Vig 2	−0.260	0.429
Inf 4	−0.261	0.452
Inf 3.3	−0.299	0.347
Inf 7	−0.404	0.407

Note: The coding of each of the items that make up the questionnaire (and thus its full text) is presented in Supplementary Materials 1 together with the Portuguese and Spanish versions of the instrument and an English translation (there is no validated English version of the EAAPE). Items Def 2 and P.G. 2 have been excluded because they present a factor load lower than 0.30 in each factor.

## Data Availability

The data presented in this study are available on request from the corresponding author.

## Supplementary Materials

The following are available online at https://www.mdpi.com/article/10.3390/nursrep12010012/s1, Table S1: Portuguese, Spanish, and English versions of EAAPE.
