# Supplementary material for: Spanish Version of the Scale “Eventos Adversos Associados às Práticas de Enfermagem” (EAAPE): Validation in Nursing Students
Source: Nurs Rep. 2022 Feb 14;12(1):112–24. doi: 10.3390/nursrep12010012 (PMC8883960; doi:10.3390/nursrep12010012)
Supplement: Supplementary file 1 [file nursrep-12-00012-s001.zip › nursrep-1547288-supplementary.pdf]

Supplementary Material Table S1. Portuguese, Spanish and English versions of EAAPE.

		PORTUGUESE VERSION (original)		SPANISH VERSION		ENGLISH TRANSLATION	
SUBSCALE	COD	CATEGORIA	ITEM	CATEGORÍA	ITEM	CATEGORY	ITEM
II	Vig. 1	Vigilância/Julgamento clínico	Os doentes são adequadamente vigiados	Vigilancia /Seguimiento clínico	Los pacientes están correctamente vigilados	Surveillance / Clinical monitoring -tracking	Patients are properly monitored
II	Vig 2		As alterações do estado clínico são oportunamente detectadas		Las alteraciones en el estado clínico son adecuadamente detectadas		Alterations in clinical status are adequately detected
I	Vig 3		Existe risco de agravamento/complicações do estado do doente por défice de vigilância		Existe riesgo de que el estado del paciente sufra complicaciones o empeoramiento por déficit de vigilancia		There is a risk that the patient's condition may suffer complications or worsen due to a lack of surveillance
I	Vig 4		Existe risco de agravamento/complicações do estado do doente por julgamento clínico inadequado		Existe riesgo de agravamiento/complicaciones del estado del paciente por seguimiento clínico inadecuado		There is a risk of worsening / complications of the patient's condition due to inadequate clinical monitoring
II	Def 1	Advocacia	Os enfermeiros assumem-se como verdadeiros "advogados" dos interesses do doente e família	Defensa de los derechos del paciente	Las/los enfermeras/os se erigen como verdaderas/os "defensores" de los intereses del paciente y la familia	Defense of patient rights	Nurses stand as true "defenders" of the patients and families' interests
-	Def 2		Os enfermeiros questionam a prática de outros		Las/los enfermeras/os cuestionan la práctica de otros profesionales cuando		Nurses question other professionals' practice

			profissionais quando está em causa o interesse do doente		está en juego el interés del paciente		when the interest of the patient is at stake
II	Def 3		Os enfermeiros respeitam a privacidade do doente		Las/los enfermeras/os respetan la privacidad del paciente		Nurses respect patient privacy
II	Def 4		Os enfermeiros respeitam a confidencialidade do doente		Las/los enfermeras/os respetan la confidencialidad del paciente		Nurses respect patient confidentiality
I	Def 5		Os enfermeiros delegam funções de enfermagem noutros profissionais menos preparados		Las/los enfermeras/os delegan funciones de enfermería en otros profesionales menos preparados		Nurses delegate nursing functions to other less trained professionals
I	Def 6		Existe risco de agravamento/complicações no estado do doente por falhas na defesa dos interesses do doente.		Existe riesgo de agravamiento/complicaciones del estado del paciente por falta de defensa de los intereses del paciente		There is a risk of aggravation/complications of the patient's condition due to lack of defense of the patient's interests
I	Def 7		Existe risco de agravamento/complicações no estado do doente por delegação de funções de enfermagem em pessoal menos preparado		Existe riesgo de agravamiento/complicaciones del estado del paciente por la delegación de funciones de enfermería en personal menos preparado		There is a risk of aggravation/complications of the patient's condition due to the delegation of nursing functions to less-trained professionals
II	Ca 1	Quedas	O risco de quedas é avaliado em todos os doentes, de acordo com protocolo instituído.	Caídas	El riesgo de caídas es evaluado en todos los pacientes de acuerdo con el protocolo establecido.	Falls	According to the established protocol, the risk of falls is evaluated in all patients.

II	Ca 2		Os procedimentos de prevenção de quedas são ajustados tendem em consideração a avaliação do risco		Los procedimientos de prevención de caídas se ajustan teniendo en cuenta la evaluación del riesgo.		Fall prevention procedures are adjusted with the risk assessment in mind.
II	Ca 3		A vigilância do doente é ajustada ao risco avaliado		La vigilancia del paciente se ajusta al riesgo evaluado		Patient surveillance is adjusted to the assessed risk
I	Ca 4		Existe risco de ocorrência de quedas de doentes		Existe riesgo de caídas en los pacientes		There is a risk of falls in patients
I	Ca 5		Ocorrem quedas de doentes		Se producen caídas de los pacientes.		Falls of patients occur
II	Ulc 1	Úlceras de pressão	No início do internamento é realizada uma avaliação clínica global (grau de mobilidade, incontinência urinária/fecal, alterações da sensibilidade, alterações do estado de consciência, doença vascular, estado nutricional).	Úlceras por presión	Al inicio del ingreso se realiza una evaluación clínica global (grado de movilidad, incontinencia urinaria/fecal, alteraciones de la sensibilidad, alteraciones del estado de conciencia, patología vascular, estado nutricional).	Pressure ulcers	At the beginning of the hospital admission, a global clinical evaluation is carried out (degree of mobility, urinary / fecal incontinence, alterations in sensitivity, altered state of consciousness, vascular pathology, nutritional status).
II	Ulc 2		É realizada a inspecção periódica da pele em áreas de risco ou de úlceras prévias		Se realiza la inspección periódica de la piel en zonas de riesgo o de úlceras previas.		Periodic skin inspection is carried out in areas of risk or previous ulcers.
II	Ulc 3		São utilizadas escalas de estratificação do risco (escalas de Braden e/ou de Norton)		Se utilizan escalas de estratificación de riesgo (escalas de Braden y/o de Norton).		Risk stratification scales are used (Braden and / or Norton scales).

II	Ulc 4		São implementadas medidas preventivas ajustadas aos factores de risco		Se implementan medidas preventivas ajustadas a los factores de riesgo.		Preventive measures adjusted to risk factors are implemented.
II	Ulc 5		Os cuidados gerais à pele são adequados às necessidades identificadas		Los cuidados generales de la piel se adecúan a las necesidades identificadas.		General skin care is tailored to the identified needs.
II	Ulc 6		O suporte nutricional é ajustado às necessidades		El soporte nutricional se ajusta a las necesidades.		Nutritional support is tailored to need.
II	Ulc 7		Os posicionamentos são ajustados às necessidades		Las posiciones/movilizaciones se ajustan a las necesidades de la persona		Positions / mobilizations are adjusted to the patient's needs
I	Ulc 8		Existe o risco de ocorrência de úlceras de pressão		Existe riesgo de aparición de úlceras por presión.		There is a risk of the pressure ulcers' onset.
I	Ulc 9		Ocorrem úlceras de pressão		Aparecen úlceras por presión.		Pressure ulcers appear.
I	Med 1	Medicação	Existe o risco de ocorrência de erros de medicação	Medicación	Existe el riesgo de aparición de errores en la medicación.	Medication	There is a risk of medication errors.
I	Med 2		Ocorrem erros de medicação		Se producen errores en la medicación		Medication errors occur
			Ocorrem erros na preparação da medicação por		Se producen errores en la preparación de la medicación por:		Errors occur in the preparation of medication due to:
I	Med 3.1		Existirem medicamentos com rótulo e embalagem semelhantes		Existencia de medicamentos con nombre o envase semejantes		Existence of drugs with a similar name or packaging
I	Med 3.2		Existirem muitos medicamentos no mesmo horário		Hay muchos medicamentos pautados en el mismo horario.		There are many medications prescribed at the same time.

I	Med 3.3	A farmácia enviar o medicamento errado	El servicio de farmacia envía un medicamento equivocado.	The pharmacy service sends the wrong medicine.
I	Med 3.4	O medicamento não estar disponível em tempo oportuno	El medicamento no está disponible en el momento oportuno.	The drug is not available when appropiate
I	Med 3.5	O enfermeiro ser interrompido durante a actividade	La enfermera/el enfermero es interrumpida/o durante la actividad.	The nurse is interrupted during the activity.
I	Med 3.6	Distracção do enfermeiro	Distracción de la enfermera/del enfermero.	Nurse distraction.
		Ocorrem erros na administração da medicação por:	Se producen errores en la administración de fármacos por:	Errors in drug administration occur due to:
I	Med 4.1	Falhas na comunicação sobre mudanças na acomodação dos doentes (troca de cama)	Fallos en la comunicación sobre cambios en la ubicación de los pacientes (cambio de cama)	Failure to communicate about changes in patient location (bed change)
I	Med 4.2	Falhas na comunicação médico/enfermeiro sobre alterações na prescrição médica	Fallos en la comunicación medico/a-enfermera/o sobre modificaciones en la prescripción	Failures in doctor-nurse communication or about prescription modifications
I	Med 4.3	Falhas na comunicação (prescrição médica oral ou por telefone)	Fallos en la comunicación (prescripción médica oral o por teléfono)	Communication failures (oral or telephone prescription)

I	Med 4.4		Falhas na comunicação (ausência de registo da administração anterior)		Fallos en la comunicación (fallo de registro de la administración anterior)		Communication failures (previous administration registration failure)
I	Med 4.5		Incorrecta identificação do medicamento preparado		Incorrecta identificación del medicamento preparado.		Incorrect identification of the prepared medicine.
I	Med 4.6		Incumprimento dos procedimentos de identificação do doente		Incumplimiento de los procedimientos de identificación del paciente.		Breach of patient identification procedures.
I	Med 4.7		Falhas na execução da técnica de administração		Fallos en la ejecución de la técnica de administración.		Failures in the execution of the administration technique.
			Vigilância da medicação		Control de la medicación		Medication control
I	Med 5.1		Ocorrem falhas na vigilância dos ritmos das perfusões		Se producen errores en la vigilancia de los ritmos de perfusión.		Errors occur in monitoring perfusion rhythms.
I	Med 5.2		Ocorrem falhas na vigilância dos efeitos da medicação		Se producen errores en la vigilancia de los efectos de la medicación.		Errors occur in monitoring the effects of medication.
I	Inf 1	Infecção associada aos cuidados de saúde (IACS)	Existe risco de ocorrerem infecções (IACS)	Infección asociada a los cuidados de salud (IACS)	Existen riesgos de que se produzcan infecciones	Healthcare associated infection (HAIs)	There are risks of infection
I	Inf 2		Ocorrem infecções (IACS)		Se producen infecciones asociadas a los cuidados de salud.		Infections associated with health care occur.
			A Higienização das mãos realiza-se:		El lavado de manos se realiza		Hand washing is done:

II	Inf 3.1	Antes e após o contacto com o doente	Antes y después del contacto con el paciente.	Before and after contact with the patient.
II	Inf 3.2	Antes de procedimentos que exijam assepsia	Antes de procedimientos que exijan asepsia.	Before procedures that require asepsis.
II	Inf 3.3	Após o contacto com sangue e fluidos corporais	Después del contacto con sangre o fluidos corporales.	After contact with blood or body fluids.
II	Inf 4	Os Equipamento de Protecção Individual (EPI) são seleccionados e ajustados aos procedimentos a realizar	Los Equipos de Protección Individual (EPI) se seleccionan y ajustan a los procedimientos a realizar.	The Personal Protective Equipment (PPE) is selected and adjusted to the procedures carried out.
II	Inf 5	Na manipulação de material corto/perfurante são evitados procedimentos inadequados, nomeadamente dobrar ou recapsular agulhas, após a sua utilização	En la manipulación del material cortopunzante se evitan procedimientos inadecuados como doblar o recapsular agujas, tras su utilización.	When handling medical sharps, inappropriate procedures such as bending or encapsulating needles are avoided after use.
II	Inf 6	Os objectos cortam/perfurantes (agulhas, lâminas de bisturi, etc.) são acondicionados em contentores rígidos, localizados próximo da realização do procedimento	Los objetos cortopunzantes (agujas, hojas de bisturí, etc.) son depositados en contenedores rígidos, localizados cerca del lugar de utilización.	Sharps (needles, scalpel blades, etc.) are deposited in rigid containers near the place of use.
II	Inf 7	A acomodação dos doentes realiza-se de acordo com a susceptibilidade imunológica e condição clínica do doente (ex.	La ubicación de los pacientes se realiza de acuerdo con la susceptibilidad inmunológica y condición clínica del paciente (ej.	The placement of patients is made according to the immunological susceptibility and clinical condition of the patient

			isolamento de acordo com as necessidades)		Aislamiento de acuerdo con las necesidades)		(e.g., isolation according to needs)
II	Inf 8		Os resíduos hospitalares são objecto de tratamento apropriado, consoante o grupo a que pertencem		Los residuos hospitalarios son objeto del tratamiento apropiado, en función del grupo al que pertenece.		Hospital waste is subject to appropriate treatment, depending on the group to which it belongs.
II	Inf 9		A roupa suja é triada junto do local de proveniência, acondicionada em saco próprio e transportada para a lavandaria em carro fechado		La ropa sucia es clasificada en el lugar de procedencia, acondicionada en bolsa apropiada y transportada a la lavandería en carro cerrado.		Dirty clothes are classified at origin, packed in an appropriate bag, and transported to the laundry in a closed cart.
I	P.G. 1	Percepção Geral (no meu serviço)	A ocorrência de eventos adversos associados às práticas de enfermagem compromete a segurança do doente	Percepción General (en mi servicio)	La aparición de eventos adversos asociados a la práctica de enfermería compromete la seguridad del paciente.	General perception	The appearance of adverse events associated with nursing practice compromises patient safety.
-	P.G. 2		Os eventos adversos associados às práticas de enfermagem podiam ser evitados		Los eventos adversos asociados a la práctica pueden ser evitados.		Adverse events associated with the practice can be avoided.
